# Proactive Control Mediates the Relationship Between Working Memory and Math Ability in Early Childhood

**DOI:** 10.3389/fpsyg.2021.611429

**Published:** 2021-05-07

**Authors:** Chunjie Wang, Baoming Li, Yuan Yao

**Affiliations:** ^1^Institute of Brain Science and Department of Psychology, School of Education, Hangzhou Normal University, Hangzhou, China; ^2^Department of Psychology, Suzhou University of Science and Technology, Suzhou, China

**Keywords:** proactive control, working memory, math ability, individual differences, early childhood

## Abstract

Based on the dual mechanisms of control (DMC) theory, there are two distinct mechanisms of cognitive control, proactive and reactive control. Importantly, accumulating evidence indicates that there is a developmental shift from predominantly using reactive control to proactive control during childhood, and the engagement of proactive control emerges as early as 5–7 years old. However, less is known about whether and how proactive control at this early age stage is associated with children’s other cognitive abilities such as working memory and math ability. To address this issue, the current study recruited 98 Chinese children under 5–7 years old. Among them, a total of 81 children (mean age = 6.29 years) contributed useable data for the assessments of cognitive control, working memory, and math ability. The results revealed that children at this age period predominantly employed a pattern of proactive control during an AX-Continuous Performance Task (AX-CPT). Moreover, the proactive control index estimated by this task was positively associated with both working memory and math performance. Further regression analysis showed that proactive control accounted for significant additional variance in predicting math performance after controlling for working memory. Most interestingly, mediation analysis showed that proactive control significantly mediated the association between working memory and math performance. This suggests that as working memory increases so does proactive control, which may in turn improve math ability in early childhood. Our findings may have important implications for educational practice.

## Introduction

Cognitive control, the ability to regulate and coordinate goal-directed behavior so as to allow for flexible adaptation to changing environments, has been considered as one of the most basic cognitive skills in humans (Miller and Cohen, 2001). Previous research has indicated that cognitive control is involved in a wide range of cognitive activities including learning (Abrahamse et al., 2016), comprehension (Ye and Zhou, 2008), theory of mind (Carlson and Moses, 2001), problem solving (Passolunghi and Siegel, 2001), and general fluid intelligence (Benedek et al., 2014). Moreover, measures of cognitive control have been shown to explain a significant amount of variance in academic achievements, above and beyond the effect of general fluid intelligence (Magalhães et al., 2020). Given these critical aspects, the numbers of studies investigating cognitive control have increased dramatically during the past decades. However, to date, much of the prior research has focused on the individual executive skills such as inhibition control, working memory, and mental-set shifting through which cognitive control is exerted (Diamond, 2013; Morra et al., 2018). Relatively few research efforts have been dedicated to examine the temporal dynamics of how cognitive control is used.

Importantly, a recently developed cognitive theory, the dual mechanisms of control (DMC) model, proposes that cognitive control can be implemented with two temporally distinct cognitive processes: proactive control and reactive control, and humans can flexibly shift between these two cognitive control processes for high-order cognition (Braver, 2012). Proactive control refers to the cue-driven and top-down cognitive processes that can allow an individual to maintain task-relevant goals in advance of the stimuli requiring a response. Reactive control, on the other hand, refers to the probe-driven and bottom-up cognitive processes in which relevant information cannot be utilized until an event requiring a response has occurred (Braver et al., 2007). Generally, these two types of cognitive control processes can be assessed using a specific experimental paradigm, the AX-Continuous Performance Task (AX-CPT, Braver et al., 2007). In AX-CPT, cue-probe pairs are presented sequentially. Participants are instructed to make a target response to the probe when an A cue is followed by an X probe (AX trials), and to make a non-target response for all other cue-probe pairs including AY, BX, and BY trials, where Y and B represent any stimuli other than A and X. Considering the high proportion of AX trials during this task, participants who use a proactive form of cognitive control tend to prepare a target response when an A cue appears. Hence, they are inclined to prepare an incorrect target response when an A cue is not followed by an X probe (AY trials). Moreover, the participants are inclined to prepare a correct non-target response when a B cue appears, even if it is followed by an X probe (BX trials). By contrast, participants who use a reactive form of cognitive control do not prepare a response according to the cue presented. Thus, they do not need to overcome the strong target expectancy that an A cue is followed by an X probe, and should make a correct non-target response on AY trials quickly. However, the X probe tends to lure them into incorrect target responses on BX trials. Nowadays, the AX-CPT has been widely used to examine proactive and reactive control in adults, repeatedly showing that young adults rely more on proactive control with worse performance on AY than BX trials (Braver et al., 2007), whereas older adults demonstrate a typical reactive pattern with worse performance on BX than AY trials (Paxton et al., 2008; Braver et al., 2009).

Several recent studies have tried to examine proactive and reactive control in children, and propose that age-related improvements in cognitive control during childhood may be accounted for by a developmental shift from heavy reliance on reactive control to more proactive control (Brahmbhatt et al., 2010; Munakata et al., 2012; Lucenet and Blaye, 2014; Chevalier et al., 2015; Troller-Renfree et al., 2020). In this view, younger children tend to rely almost exclusively on reactive control, a late correction mechanism that involves waiting for a control-demanding event to occur and then implements cognitive control in a just-in-time manner. Conversely, older children can use both forms of cognitive control. As age increases, they tend to rely more on proactive control, through which they could actively maintain goal-relevant information before an event occurs and thereby optimally orient behavior. Compared with reactive control, proactive control poses a greater cognitive demand on working memory, but it is generally more effective, which may explain better behavioral performance in many cognitive skills (Chevalier et al., 2013; Gonthier et al., 2019). To date, the efficiency in proactive control during childhood has been convincingly shown to increase with age, with older children demonstrating more and more advantages on BX than AY trials (Chatham et al., 2009; Lorsbach and Reimer, 2010). Moreover, recent work suggests that the shift from reactive to proactive control begins in early childhood – presumably occur at around 5–7 years of age (Lucenet and Blaye, 2014; Gonthier et al., 2019).

However, it remains unclear whether proactive control in early childhood is associated with other cognitive abilities. The literature has put one possible answer forward: working memory. Critically, working memory requires individuals to actively maintain and manipulate task-related information, and proactive control requires individuals to use proactive cues to prepare for maintaining and manipulating task-related information (Braver, 2012). In addition, neuroimaging studies have consistently reported that proactive control recruits brain regions (e.g., the prefrontal cortex) that are largely overlapping with the working memory network (Müller and Knight, 2006; Aron, 2011). Hence, it is reasonable to speculate that working memory may be related to the use of proactive control. In agreement with this hypothesis, accumulating evidence has demonstrated proactive control is closely related to working memory in adults (Redick, 2014; Richmond et al., 2015; Wiemers and Redick, 2018). For instance, Redick (2014) showed that in young adults, individuals with high working memory capacity tend to use proactive control more often than individuals with low working memory capacity. Additionally, Richmond et al. (2015) reported that in young adults, inter-individual differences in working memory could predict inter-individual differences in the efficiency of proactive control engagement. A few studies also reported similar relationships during childhood (Lorsbach and Reimer, 2010; Troller-Renfree et al., 2020). For instance, individual differences in working memory were found positively related to more proactive control in children at 9 years old (Troller-Renfree et al., 2020). Given the positive relationship between working memory and proactive control reported in both adulthood and late childhood, we hypothesize that working memory may relate to the use of proactive control in early childhood. Investigation of this question would bring us a deeper understanding of the mechanisms underlying cognitive development during early childhood.

A related question is whether proactive control in early childhood could be linked to academic abilities. Of particular interest is the math ability that is critical to many aspects in daily life (Hafer et al., 2002; Shapka et al., 2006; Joensen and Nielsen, 2009). Research has documented numerous cognitive factors that may affect math ability (Clark et al., 2010; Raghubar et al., 2010). One of the most investigated factors is working memory (Raghubar et al., 2010). It is assumed that operational processes in math problem solving involve temporary storage and retrieval of task-relevant information, which greatly consumes working memory resources. Since proactive control and working memory have been suggested to share overlapping cognitive processes and neural resources (Müller and Knight, 2006; Aron, 2011; Braver, 2012; Redick, 2014), the use of proactive control at early childhood may play a critical role in the development of math ability, and even affect the impact of working memory on math ability. To date, only one study specifically focused on the relationship between proactive control and math ability in children, and reported that individual differences in proactive control engagement were positively related to variations in math performance (Kubota et al., 2020). Notably, this relationship was detected in a sample with a wide age range of 6–10 years old, and most of the participants were in middle or late childhood. It remains unclear whether the use of proactive control in early childhood contributes to individual differences in math performance.

Based on the literature mentioned above, the present study aimed to investigate the relationships of proactive control with both working memory and math ability in early childhood. Of particularly, we focused on the age of 5–7 years old because this age period has been suggested as the earliest stage for the emergence of proactive control (Lucenet and Blaye, 2014; Gonthier et al., 2019). Additionally, the testing point in this study was set at the start of primary school. This is a period of interest because it is marked by important changes to both children’s cognitive abilities and external demands of the school environment. Investigating relations in cognitive abilities at this age stage can help us identify the early skills that may have long-term consequences for later cognitive and academic outcomes (Mazzocco and Kover, 2007; De Smedt et al., 2009). Children at this age period performed an animal version of the AX-CPT task that could measure engagement in proactive control. In addition, they completed several cognitive tasks that could measure working memory and math ability. Based on findings in prior research (Lucenet and Blaye, 2014; Gonthier et al., 2019), we hypothesized that children at this age period would show a predominantly proactive pattern of cognitive performance, with worse performance on AY trials than on BX trials in the AX-CPT. Besides, we hypothesized that the proactive control index as measured by the AX-CPT would be positively correlated with both working memory and math performance. Moreover, since prior research has consistently reported that working memory is closely related to math performance (Raghubar et al., 2010), we further tested whether the relationship between proactive control and math performance would be independent from the relationship between working memory and math performance, and whether individual differences in proactive control would mediate the relationship between working memory and math performance. Additionally, the literature has provided some evidence that variations in age, gender, socioeconomic status, and fluid intelligence of the samples may affect behavioral performance in working memory and math ability during childhood (Espy et al., 2004; Noble et al., 2007; Wei et al., 2012; Yeniad et al., 2013). Therefore, these variables would be incorporated into the present study as control variables.

## Materials and Methods

### Participants and Procedures

We enrolled a total of 98 children aged 5–7 years old from a primary school in Chinese Mainland. They were from rural families, had normal hearing and normal visual acuity, had no history of psychiatric or neurological disorders, and had studied the same curriculum with no special educational assistance requirements. At the time of testing, they were all at the beginning of their first-grade years. Specifically, three computer-based cognitive tasks generated using E-Prime 1.1 were used to measure proactive control, and verbal/visual working memory. The children were tested one-by-one in a quiet room at school, and the order of the three computerized tasks was counterbalanced between subjects. After all the computerized tasks, two paper-pencil tests that assessed math and fluid intelligence were administrated in a group manner. Additionally, parents finished a questionnaire including a widely used marker of socioeconomic status – average monthly household income. A total of 15 children were not included in final statistical analyses due to withdrawal from the study after the enrollment (*N* = 9) or incomplete behavioral assessments (*N* = 6). Moreover, two children were excluded due to the mean of accuracy in the AX-CPT task being below 50% and 3 SD below the mean accuracy of all participants. Consequently, 81 children constituted the final analytical sample (*N* = 81, mean age = 6.29 years, SD = 0.35, range = 5.76–7.32, 40 boys, Table 1).

**Table 1:** Mean (SD) and range of all the study variables (*N* = 81).

Study variables		Mean	SD
Age		6.29	0.36
Gender	Percentage of boys	49.4%	
**Socioeconomic status**			
Monthly household income (RMB)		5,360	2,711
Fluid intelligence	Raw scores	16.35	4.46
**AX-CPT**			
Reaction time	AX	584	90
	AY	800	129
	BX	662	139
	BY	702	130
Error rates	AX	0.18	0.08
	AY	0.38	0.14
	BX	0.27	0.13
	BY	0.14	0.09
Proactive control indices	PBI in reaction time	0.10	0.10
	PBI in error rates	0.17	0.35
	Composite PBI	0.00	0.76
**Working memory**			
Verbal working memory		5.76	0.94
Visual working memory		4.79	1.05
Composite working memory		0.00	0.83
Math		42.35	11.53
Number copying		20.22	6.94

SD, standard deviation.

### Cognitive Control

An animal version of the AX-CPT task was used to measure cognitive control (Gonthier et al., 2019). In this task, if an X probe (giraffe) occurred after an A cue (panda), participants were instructed to press the green button with their dominant index finger, but if any other cue-probe pairs (AY, BX, and BY trials, where Y and B represent any animals other than panda and giraffe) occurred, participants were required to press the red button with their other index finger. They were instructed to respond as fast and accurately as possible. Similar to prior research (Lucenet and Blaye, 2014), AX trials made up 70% of the trials, while each of the other three types of cue-probe pairs made up 10% of the trials. There were 16 practice trials, which could be repeated one more time if needed, to make the participants acquainted with this task. The formal testing included four blocks of 80 trials, yielding a total of 320 trials. For each trial, a fixation was firstly displayed in the center of the screen for 500 ms; then a cue animal picture was presented on the screen for 500 ms, followed by a blank interval for 1,500 ms; subsequently, a probe animal picture was presented on the screen up to 1,500 ms or disappeared if a response was given. Error rates and mean reaction time for correct responses were calculated for each condition. Then the proactive behavioral index (PBI) that has been used widely in previous research (Gonthier et al., 2019; Kubota et al., 2020), was calculated to measure the use of proactive control. The PBI score was computed as (AY − BX)/(AY + BX) for both error rates and reaction time. This index could reflect the relative balance of interference between AY and BX trials, where a higher value in PBI scores would reflect more reliance on proactive control.

### Working Memory

Both verbal and visual working memory tasks were conducted to obtain a domain-general estimate of working memory ability.

Verbal working memory was measured by a forward digit memory span task that was derived from the Wechsler intelligence scale (Watkins and Smith, 2013). In this task, a set of sequences with single digits (1–9) were presented aurally at a rate of one digit per second. Participants were instructed to repeat those numbers in order immediately after the presentation of the last digit. The task started with a sequence length of two digits, and each length was tested with two independent digit sequences. The sequence length would increase by 1 if either one or both digit sequences for the same length were recalled correctly, otherwise the task would be discontinued. Verbal working memory was determined by the maximum sequence length the subject could recall correctly. If both trials of the maximum sequence length were recalled successfully, verbal working memory was indexed by this sequence length; otherwise verbal working memory was indexed by the maximum length minus 0.5.

Visual working memory was measured by an animal span task adapted from Loosli et al. (2012). The task consisted of two stages. In the encoding stage, a sequence of animals was presented in the center of the screen and participants were asked to identify the orientation of each animal by pressing the left or right button (press the right button for correct presentation and press the left button for upside-down). At the same time, they were required to remember the order in which the animals were presented. If the participants made a wrong response or did not give a response within 3,000 ms, an error feedback was presented. In the recall stage, the participants were required to recall the previously displayed animal sequence by clicking on the appropriate animals from the display without time limits. The task started with a sequence length of two animal pictures. The length on the next animal sequence would increase by 1 if the participant recalled the current animal sequence correctly, otherwise it would remain stable. The task would be discontinued if the participants could not correctly recall two animal sequences with a same length. Visual working memory was assessed by the maximum number of animals that the participant could recall correctly.

### Math

Math ability was measured by the arithmetic subscale of the Heidelberg Rechentest (Haffner, 2005), which has been reported to have good reliability for the Chinese population (Wu and Li, 2005). It consisted of four timed subtests: mental addition (e.g., 7+1=_), mental subtraction (e.g., 60−4=_), number equations filling (e.g., 11+_=15–2), and number comparison (e.g., 12+9_20). Problems in each subscale were displayed serially in a list with an order of increasing difficulty. Participants were instructed to solve the math problems with numbers or symbols such as “>,” “<,” and “=” within a time limit of 1 min for each subtest. For each participant, the number of correct answers combined for all subtests was used as an estimate of math ability. Moreover, this math test provides an additional subtest for number writing speed. Children were required to copy as many numbers as possible within 30 s. This measure could be used to control for the effect of general writing speed on math performance.

### Fluid Intelligence

Fluid intelligence was measured by Raven’s Standard Progressive Matrices (Raven, 2003). The test has also been reported to show good reliability for the Chinese population (Wang et al., 2007). It includes 72 items and each item consists of a series of geometric figures with one of them missing. Participants were asked to choose the appropriate geometric figure from a set of given figures. To reduce fatigue in the child participants, we split the Raven’s test into odd and even items (36 items per version), and only the version with odd items was used. Participants had 20 min to complete the test, and the number of correct responses was used as a measure of fluid intelligence.

## Result

### Descriptive Analyses

In the AX-CPT task, the mean of reaction time and error rates across all trials were 625 ms (SD = 89, range = 416–945) and 0.21 (SD = 0.06, range = 0.06–0.34), respectively. Mean reaction time was not significantly correlated with error rates (*r* = 0.012, *p* = 0.918), indicating no speed-accuracy trade-off in AX-CPT. Consistent with previous studies (Lucenet and Blaye, 2014; Kubota et al., 2020), the children performed more slowly [*t*(80) = 8.195, *p* < 0.001, *d* = 152 ms] and committed more errors [*t*(80) = 4.613, *p* < 0.001, *d* = 0.107] on the AY trials than the BX trials, thereby revealing their use of a proactive mode of cognitive control. To estimate the inter-individual differences in the use of proactive control, the PBI scores in term of both reaction time and error rates were computed for each participant. A higher value would indicate more use in proactive control processes. Then a composite PBI score was computed by standardizing and averaging the reaction time PBI scores and error rates PBI scores, thereby summarizing the use of proactive control with a single index. Regarding to the two working memory measures, verbal working memory was found positively related to visual working memory (*r* = 0.392, *p* < 0.001). Then a composite working memory score was computed by standardizing and averaging the scores on these two working memory tasks so as to obtain a domain-general estimate of working memory capacity. The descriptive statistics of all the measures in the present study are displayed in Table 1.

### Correlational Analyses

We first examined the potential relations of age, socioeconomic status, fluid intelligence, and number copying speed with our key study variables. As shown in Table 2, significant correlations were detected between fluid intelligence and composite PBI scores (*r* = 0.228, *p* = 0.041), between fluid intelligence and visual working memory (*r* = 0.236, *p* = 0.034), as well as between fluid intelligence and math ability (*r* = 0.224, *p* = 0.045). No other significant relationships with these key study variables were detected. Additionally, none of these key study variables showed any significant gender differences (smallest *p* = 0.152).

**Table 2:** The correlations of age, socioeconomic status, and fluid intelligence with key study variables.

	Age	Socioeconomic status	Intelligence	Number copying
**Proactive control**				
PBI _reaction time_	−0.061	−0.083	0.194	0.082
PBI _error rates_	0.008	−0.077	0.151	−0.031
Composite PBI	−0.035	−0.106	0.228^*^	0.034
**Working memory**				
Verbal	0.031	−0.164	−0.002	0.099
Visual	−0.081	−0.16	0.236^*^	0.048
Composite	−0.030	−0.194	0.14	0.088
Math	0.114	−0.095	0.224^*^	0.106

* *p* < 0.05.

**Figure 1:**
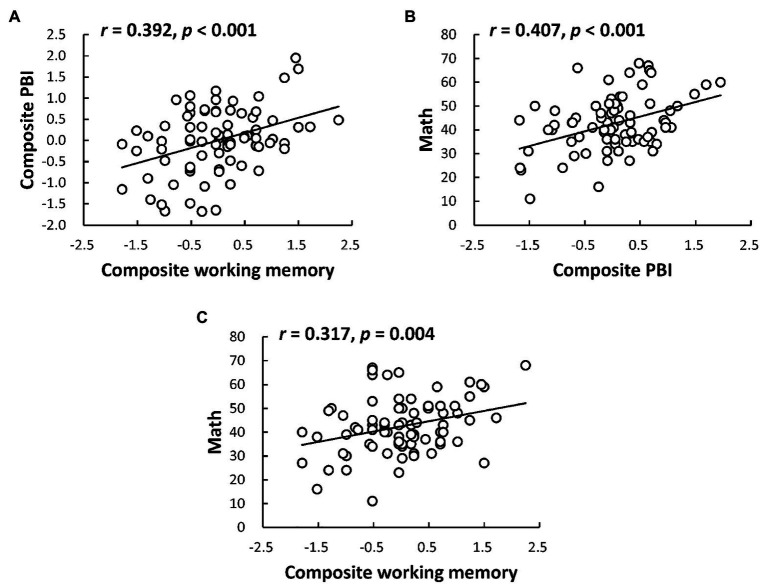
Relationships among proactive control, working memory, and math ability. **(A)** Represents the relationship between proactive control and working memory; **(B)** represents the relationship between proactive control and math ability; and **(C)** represents the relationship between working memory and math ability.

Then we examined the relations of proactive control with both working memory and math ability (Table 3). Importantly, both the composite PBI scores (*r* = 0.392, *p* < 0.001, Figure 1A) as well as PBI scores in reaction time (*r* = 0.330, *p* = 0.003) and error rates (*r* = 0.264, *p* = 0.017) were found positively correlated with the composite working memory scores, confirming that children with higher working memory capacity tended to be more proactive. To confirm the stability of this finding, the same analyses were replicated by considering the two working memory tasks separately. Significant or marginal correlations with the PBI scores were also found separately for verbal working memory (composite PBI: *r* = 0.304, *p* = 0.006; PBI _reaction time_: *r* = 0.259, *p* = 0.020; PBI _error rates_: *r* = 0.203, *p* = 0.069) and visual working memory (composite PBI: *r* = 0.349, *p* = 0.001; PBI _reaction time_: *r* = 0.292, *p* = 0.008; PBI _error rates_: *r* = 0.238, *p* = 0.032), confirming that the relationship between proactive control and working memory was domain general. Moreover, the composite PBI scores were found positively correlated with children’s math performance (*r* = 0.407, *p* < 0.001, Figure 1B). Similar correlations were also found between PBI scores in reaction time and math ability (*r* = 0.325, *p* = 0.003), as well as between PBI scores in error rates and math ability (*r* = 0.292, *p* = 0.008), providing further evidence that proactive shift of cognitive control may be beneficial for math problems solving. Notably, all the above significant correlations remained similar when controlling for the effect of fluid intelligence as well as other factors including age, socioeconomic status, and number copying speed. Additionally, in line with prior research (Raghubar et al., 2010), individual differences in composite working memory were found positively correlated with children’s math performance (*r* = 0.317, *p* = 0.004, Figure 1C). Significant correlations were also found between verbal working memory and math performance (*r* = 0.219, *p* = 0.049), as well as between visual working memory and math performance (*r* = 0.311, *p* = 0.005), indicating that the relationship between working memory and math performance was domain general. *Post hoc* power analysis using the G-Power Analysis software program (Faul et al., 2007) revealed that with the current sample size of *N* = 81, and for the observed correlations of the composite PBI scores with both working memory and math performance, the achieved power could reached the standard threshold of 0.80.

**Table 3:** The correlations of proactive control with both working memory and math ability.

	Working memory			Math
	Composite	Verbal	Visual	
**Proactive control**				
Composite PBI	0.392^***^	0.304^**^	0.349^**^	0.407^***^
PBI _reaction time_	0.330^**^	0.259^*^	0.292^**^	0.325^**^
PBI _error rates_	0.264^*^	0.203	0.238^*^	0.292^**^

*** *p* < 0.001;

** *p* < 0.01;

* *p* < 0.05.

### Regression and Mediation Analyses

Hierarchical regression analyses were carried out to examine whether proactive control could explain a significant amount of variance in math performance beyond the effect of working memory. As there was a significant correlation between fluid intelligence and math performance, the intelligence score was entered into the model as a covariate in step 1. The composite working memory score was entered into the model in step 2 to control for the effect of working memory. Finally, the composite PBI scores were entered into the model to determine the unique influence of proactive control after controlling for the effects of both fluid intelligence and working memory. Regression results were expressed in term of R-square change (ΔR^2^) accounted for by the model and standardized regression coefficients (β) of each predictor, which were displayed in Table 4. Our results showed that this final model was significant [*F*(3,77) = 6.847, *p* < 0.001, total R^2^ = 0.211]. The composite PBI scores accounted for additional 7.7% variance increase in explaining individual differences in children’s math performance. The results remained significant when controlling for other factors including age, socioeconomic status, and number copying speed.

**Table 4:** Hierarchical regression analyses predicting math performance.

Step	Total R^2^	ΔR^2^	Fluid intelligence	Working memory	Proactive control
1	0.050	0.050	0.224^*^		
2	0.134	0.084	0.183	0.292^**^	
3	0.211	0.077	0.129	0.179	0.307^**^

** *p* < 0.01;

* *p* < 0.05.

**Figure 2:**
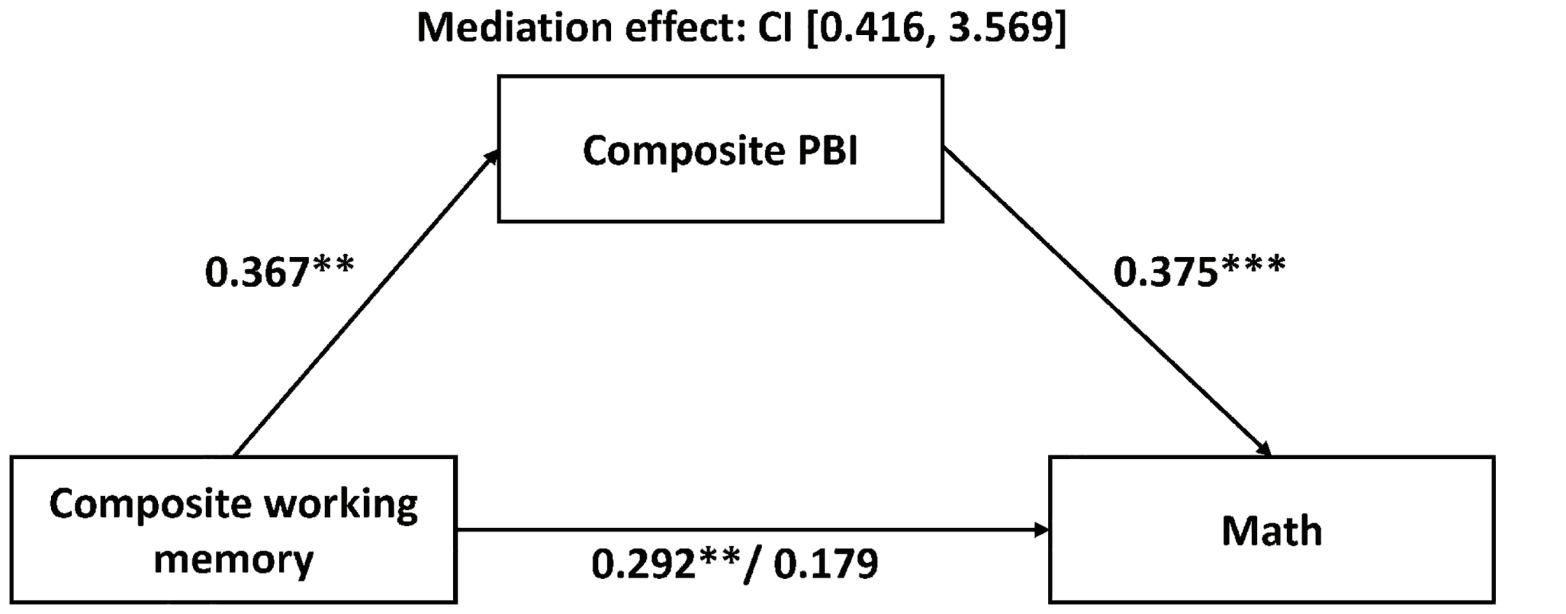
The mediation role of proactive control in the relationship between working memory and math ability. Numbers are standardized beta coefficients, and the value after the forward-slash indicates the standardized beta coefficient after the inclusion of the mediator. ^***^p < 0.001; ^**^p < 0.01.

Since proactive control, working memory, and math ability were found to be significantly correlated with each other, we further ran a mediation model to test whether individual differences in proactive control could mediate the relationship between working memory and math ability. The mediation effect was evaluated using the PROCESS (Hayes, 2012) implemented in SPSS 22.0. To test the significance of mediation effect, a 95% bootstrapped CI was generated from repeated resampling (10,000 samples) of the observed data. CIs that do not include zero would indicate a significant mediation effect of the predictor (working memory) on the outcome (math performance) through the mediator (proactive control). Given that fluid intelligence scores were found to be significantly correlated with all three measures, it was included as a covariate. The result indicated that the composite PBI scores exerted an indirect mediation effect on the relation between composite working memory and math performance [CI (0.416, 3.569), Figure 2]. The same analyses were conducted with composite working memory scores replaced by verbal or visual working memory scores, respectively. Similarly, the composite PBI scores mediated the relationship between verbal/visual working memory scores and math performance [verbal working memory: CI (0.348, 2.732); visual working memory: CI (0.253, 2.596)]. These results remained significant when controlling for age, intelligence, socioeconomic status, and number copying speed.

## Discussion

It has been now well accepted that cognitive control improves rapidly during childhood, with one of the dominant changes being a developmental shift from predominantly reactive control to a more planful and sustained pattern of proactive control (Lucenet and Blaye, 2014; Gonthier et al., 2019; Troller-Renfree et al., 2020). However, less is known about whether and how this proactive shift of cognitive control relates to other cognitive abilities. Although several recent studies have provided evidence for significant associations between the use of proactive control and individual differences in working memory and math ability, they focused on adults (Redick, 2014; Wiemers and Redick, 2018), older children (Troller-Renfree et al., 2020), or children with a wide age range (Kubota et al., 2020). It is currently not clear whether and how proactive control at early childhood relates to individual differences in working memory and math ability. The current study tried to address this question in children under 5–7 years of age. First, consistent with prior research (Lucenet and Blaye, 2014; Gonthier et al., 2019), the present study demonstrated that children aged 5–7 years old engaged cognitive control more proactively, as reflected by worse performance in term of both response time and error rates on AY than BX trials. Second, the proactive control index measured by the AX-CPT task was found positively associated with behavioral performance in both working memory and math tasks. Third, hierarchical regression analyses indicated that proactive control accounted for additional variance in predicting math ability beyond the effect of working memory. Finally, a mediation model showed that individual differences in proactive control significantly mediated the relationship between working memory and math ability. Altogether, these findings suggest that proactive control during early childhood is closely related to inter-individual differences in working memory and math ability, which may have important implications for future educational interventions.

### The Use of Proactive Control

Previous research has consistently reported that as age increases, children shift from heavy reliance on reactive control to more proactive control during childhood (Brahmbhatt et al., 2010; Munakata et al., 2012; Chevalier et al., 2013; Lucenet and Blaye, 2014; Troller-Renfree et al., 2020). Importantly, a recent study by Gonthier et al. (2019) suggests that this developmental shift begins in early childhood – presumably occur at around 5–7 years of age. In their study, pre-kindergartners (mean age = 4.41 years) showed a clear pattern of reactive control in the AX-CPT, with higher error rates on BX than AY trials. In contrast, kindergartners (mean age = 5.72 years) and first-grade children (mean age = 6.68 years) showed more reliance on proactive control, with both more errors and longer reaction time on AY than BX trials. Interestingly, our study also revealed that children aged 5–7 years old engaged cognitive control more proactively, with higher error rates and longer reaction time on AY than BX trials (Table 1). The sample age in our study was similar to the age of kindergartners and first-grade children in the study by Gonthier et al. (2019). Hence, our study, with a relatively larger sample size, replicated their findings and suggested that children aged 5–7 years old have acquired the ability to use proactive control.

### Relationships Between Working Memory and Proactive Control

Previous research has consistently reported that individual differences in working memory are significantly correlated with variations in proactive control, indicating some common cognitive substrates between these two processes (Lorsbach and Reimer, 2010; Richmond et al., 2015; Wiemers and Redick, 2018; Troller-Renfree et al., 2020). It has been suggested that the engagement of proactive control may critically depend on working memory, as proactive control requires continuous and active maintenance of goal-related information in working memory. Accordingly, individuals with higher working memory capacity may be better at using valid cues to prepare their responses to incoming targets, and show more efficiency in proactive control engagement. However, the majority of previous studies have detected this relationship in young adults (Richmond et al., 2015; Wiemers and Redick, 2018) and older children (Lorsbach and Reimer, 2010; Troller-Renfree et al., 2020). Little is known about how this relationship unfolds in early childhood. Importantly, our study extended previous findings by revealing a positive relationship between proactive control and working memory in children at 5–7 years old. Consistent with the study by Gonthier et al. (2019), our study found that both verbal and visual working memory were positively correlated with the use of proactive control. Hence, there may be a domain-general factor of working memory rather than a domain-specific working memory component that could account for the close relationship. However, the study by Gonthier et al. (2019) used a wide age range and the relationships disappeared when controlling for age. Additionally, participants in their study were recruited from different school grades including pre-kindergarten, kindergarten, and first grade. Thus, the associations between working memory and proactive control could be interfered by confounding factors such as schooling effect (Brod et al., 2017). By contrast, our study focused only on the first-grade students and all the tests were administered at the start of the first grade. Hence, our study might provide more clear evidence for the positive relationships between working memory and proactive control in early childhood. Finally, several previous studies in adults have implicated the same brain circuitry (e.g., the prefrontal and parietal regions) in both proactive control and working memory paradigms (Müller and Knight, 2006; Aron, 2011). Hence, it is possible that the relationships between proactive control and working memory observed in the current study were driven by involvement of a shared brain circuitry. Consistent with this conjecture, a previous study reported that the links between proactive control and working memory in 9-year-old children were mediated by increases in parietal activity underlying working memory (Troller-Renfree et al., 2020). However, further study is warranted to identify the neural mechanism underlying the relation between proactive control and working memory in early childhood.

### Relationships Between Proactive Control and Math Ability

Another interesting finding of the current study is that individual differences in the use of proactive control at this early age stage were positively correlated with variations in math ability. This adds a new perspective to the field by demonstrating that children may benefit from using proactive control in specific academic skills. A growing body of studies have been dedicated to investigating the potential factors accounting for individual differences in math ability (Bull and Scerif, 2001; De Smedt et al., 2009; Wang et al., 2015). However, the majority of previous studies have tried to explain individual differences in math ability by investigating the impact of specific cognitive skills such as working memory, response inhibition, and task switching (Bull and Scerif, 2001; Raghubar et al., 2010; Wang et al., 2015). Interestingly, the selection and use of appropriate strategies has also been suggested to explain part of the variability in math ability (Imbo et al., 2007; Imbo and Vandierendonck, 2007). And a recent study in adults has shown that the use of proactive control has positive influences on the strategy selection and execution when solving math problems (Hinault et al., 2017). Hence, it is possible that the use of proactive control helps children use cues to prepare appropriate math strategies in advance, and thereby contributes to their improved math performance. Notably, considering that working memory, which was found significantly correlated with the use of proactive control in the present study, has been convincingly shown to play a critical role in the development of math ability in children (De Smedt et al., 2009; Raghubar et al., 2010), one may wonder whether individual differences in working memory accounts for the relationship between proactive control and math ability. Nevertheless, this is unlikely as our hierarchical regression model showed that proactive control still explained a unique portion of math ability after controlling for those explained by working memory. Moreover, the contribution of proactive control to math ability remained significant when the intelligence score was included as a covariate, highlighting the importance of proactive control for math performance.

### Proactive Control Mediates the Relationships Between Working Memory and Math Ability

The close relationship between working memory and math ability during childhood has been supported by a growing body of research (Swanson and Sachse-Lee, 2001; Berg, 2008; Raghubar et al., 2010; Van de Weijer-Bergsma et al., 2015). Importantly, the present study found that this relationship at early childhood was mediated by individual variations in the use of proactive control. This mediation effect could be replicated when the verbal/visual working memory scores were used as the dependent variable. Together, these results indicate that working memory may contribute to the early development of math ability through the engagement of proactive control. We speculated that children with higher working memory capacity might engage proactive control more efficiently when solving math problems. As a result, they may be easier to prepare and select appropriate math strategies and thereby accomplish the math tasks in a more efficient manner. A previous neuroimaging study by Taillan et al. (2015) investigated the neural correlates of strategy selection when solving math problems, and showed greater brain activations in the right anterior cingulate cortex, dorsolateral prefrontal cortex, and angular gyrus when selecting the better math strategies. Interestingly, these brain regions were previously observed in both working memory (Owen et al., 2005) and proactive control processes (Müller and Knight, 2006; Aron, 2011). Thus, it is also possible that the shared underlying neural mechanism contributes to the mediation role of proactive control in the effect of working memory on math performance. In the present study, the relationship between working memory and math ability was completely mediated by proactive control. This complete mediation effect might be attributable to the way the math test tested in this study. Although, all the participants in the present study answered the same math problems, children with higher working memory capacity might solve the math problems more efficiently by direct retrieval and progress further through the math test. They might thus encounter increasingly difficult math problems that elicited more procedural strategies. Given that proactive control has been suggested to play a prominent role in adaptive strategy selection such as planning the order of arithmetic operations (Hinault et al., 2017), proactive control might be thus used more frequently when solving these increasingly difficult math problems and thereby exerted a complete mediation effect.

### Limitations and Future Directions

The current study had a few limitations that should be considered in future research. First, we employed only an AX-CPT task and a standardized arithmetic test for measuring proactive control and math ability. A broader measurement for proactive control and math ability is recommended for future research to improve the generalization of the findings. Additionally, as the present study only examined a set of very limited variables, it remains unclear whether other cognitive factors untested would affect the results. Future studies should also consider other cognitive measures to address the potential confounding issues more rigorously. Second, the current findings are limited to the age range under investigation. Further study is warranted to investigate whether similar relations exist during preschool, primary school, and adolescence to address the role of proactive control in cognitive development more comprehensively. Third, the current study does not allow for conclusions about the directionality of the relationships of proactive control with working memory and math ability. Future research should consider investigating the causal relationships between proactive control and cognitive functions, for example, by examining whether targeted training on proactive control could improve working memory and math ability in children. Finally, the neural correlates of proactive control in early childhood remain largely unknown. Future research should try to clarify the potential neural mechanism that may underlie the relationships among proactive control, working memory, and math ability in early childhood.

## Conclusion

To summarize, this study indicated that individual differences in proactive control at early childhood could explain variations in working memory and math ability. Moreover, individual differences in proactive control were found to explain additional variances in math ability beyond the effect of working memory, and were found to significantly mediate the association between working memory and math ability. More rigorous studies are needed to examine the causal relationships between proactive control and various cognitive functions at early childhood, and to identify the neural mechanism underlying these relationships. Lastly, it is tempting to think that targeted training on proactive control at an early age may be helpful in enhancing cognitive and academic skills in children with cognitive deficits, which deserves a further investigation.

## Data Availability Statement

Considering that data of this study are still analyzed for further research, we can not share the data publicly at the moment. The datasets generated for this study are available on request to the corresponding author.

## Ethics Statement

The Committee on Ethics and the Institutional Review Board of Zhejiang University approved the study protocol. Parental consent was given for all children.

## Author Contributions

CW collected the data, carried out the data analysis, drafted the initial manuscript, and critically revised the manuscript for important intellectual content. YY designed the study, collected the data, and contributed to interpretation of the data and revision of the manuscript. BL contributed to interpretation of the data and revision of the manuscript. All authors contributed to the article and approved the submitted version.

### Conflict of Interest

The authors declare that the research was conducted in the absence of any commercial or financial relationships that could be construed as a potential conflict of interest.
