# Corrigendum: Proactive Control Mediates the Relationship Between Working Memory and Math Ability in Early Childhood

**DOI:** 10.3389/fpsyg.2021.775003

**Published:** 2021-09-30

**Authors:** Chunjie Wang, Baoming Li, Yuan Yao

**Affiliations:** ^1^Institute of Brain Science and Department of Psychology, School of Education, Hangzhou Normal University, Hangzhou, China; ^2^Department of Psychology, Suzhou University of Science and Technology, Suzhou, China

**Keywords:** proactive control, working memory, math ability, individual differences, early childhood

In the original article, there was an error in the Funding statement as published. The correct Name for the Funder “Hangzhou Normal University (2020QDL006 and 20JYXK035)” is “the Starting Research Fund from Hangzhou Normal University (No. 2020QDL006), and the cultivation project of the province-leveled preponderant characteristic discipline in the College of Education of Hangzhou Normal University (20JYXK035)”. The corrected Funding statement is shown below.

This research was supported by grants to CW from the National Natural Science Foundation of China (32000780), the Natural Science Foundation of Zhejiang Province (Q21C090027), China Postdoctoral Science Foundation (no. 2018M630655), the Starting Research Fund from Hangzhou Normal University (no. 2020QDL006), and the cultivation project of the province-leveled preponderant characteristic discipline in the College of Education of Hangzhou Normal University (20JYXK035), and grants to YY from the Natural Science Foundation of Jiangsu Province (no. BK20190937) and Natural Science Foundation for Universities of Jiangsu Province (no. 19KJB19005).

The authors apologize for this error and state that this does not change the scientific conclusions of the article in any way. The original article has been updated.

## Publisher's Note

All claims expressed in this article are solely those of the authors and do not necessarily represent those of their affiliated organizations, or those of the publisher, the editors and the reviewers. Any product that may be evaluated in this article, or claim that may be made by its manufacturer, is not guaranteed or endorsed by the publisher.

